# A Rare Case of Fungal Burn Wound Infection Caused by *Fusarium solani* in Vietnam

**DOI:** 10.1177/2324709620912122

**Published:** 2020-05-13

**Authors:** Que Anh Tram, Nguyen Thai Ngoc Minh, Do Ngoc Anh, Nguyen Nhu Lam, Tran Ngoc Dung, Ngo Thi Minh Chau, Le Tran-Anh

**Affiliations:** 1 Vinh Hospital of Friendship General, Vinh, Nghe An, Vietnam; 2 National Hospital of Burns, Vietnam Military Medical University, Ha Noi, Vietnam; 3 Vietnam Military Medical University, Ha Noi, Vietnam; 4 Hospital 103, Vietnam Military Medical University, Ha Noi, Vietnam; 5 Hue University of Medicine and Pharmacy, Hue, Vietnam

**Keywords:** *Fusarium solani*, fusariosis, fungal wound infection, Vietnam

## Abstract

A patient with extensive burn injuries was admitted to the National Hospital of Burns in Hanoi, Vietnam, and diagnosed with fungal wound infection by histological examination of skin biopsy samples. *Fusarium solani* was isolated and identified by analysis of its morphological features and the sequence of the internal transcribed spacer region. The isolation showed in vitro resistant to fluconazole, voriconazole, itraconazole, amphotericin B, and caspofungin. Invasive fusariosis is difficult to treat due to its angioinvasive property and its lacking amenability to treatment with antifungal drugs. This infection is rare and has not been reported so far in Vietnam.

## Introduction

*Fusarium solani* constitutes a complex of many related filamentous fungi in the division *Ascomycota*. The organism occurs ubiquitously in natural environments, particularly in soil. While being primarily a plant pathogen, some species can cause disease in humans and animals. Humans can be infected with *F. solani* through inhalation of airborne conidia or via breaks in the skin due to trauma, including burn injuries.^1^ The clinical presentation of fusariosis varies significantly and may include superficial, locally invasive, and disseminated infections, depending both on the immune status of the host and the entry site of the pathogen.^2^ Most invasive infections are observed in immunosuppressed patients, and *Fusarium* may be considered as an emerging opportunist with a preferential occurrence in (sub)tropical areas.^3,4^ Keratitis and onychomycosis are the most common conditions,^2^ and in fact, *Fusarium* species are the leading cause of fungal keratitis.^5^ Due to the loss of the protective skin barrier, patients with burn injuries are prone to *F. solani* infection.^6^ In addition to local manifestations, fungemia has also been reported.^7^ The definite diagnosis of invasive fusariosis requires detection of hyphae by microscopy and isolation of the pathogen from sterile materials or blood and/or molecular methods.^8^ Invasive fusariosis is difficult to treat due to its angioinvasive potential and poor response to antifungal therapy.^9^

Vietnam is a tropical country, so local people are at a higher risk of acquiring fusariosis than those living in regions with temperate climates.^3,4^ Another important risk factor for *Fusarium* infection is that Vietnam is still a developing country, so Vietnamese people are at a higher risk of burns than those living in developed countries,^10^ and burn patients are the subpopulation who are most commonly affected by *Fusarium* among immunocompetent hosts.^11^ However, there have been no reports of fusariosis in Vietnam.

## Case Presentation

**Figure 1.**
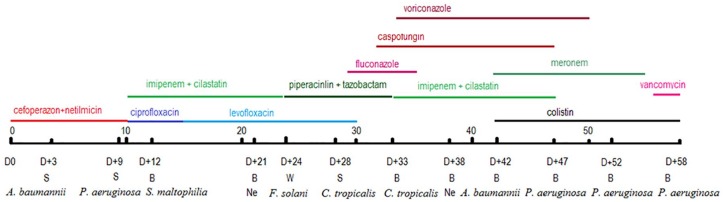
The patient’s course of infection and antibiotics/antifungal drugs. Abbreviations: B, blood; D, day; Ne, negative; S, swabs; W, wound biopsy.

A 24-year-old otherwise healthy male patient had acquired extensive burn injuries at a gasoline fire and was admitted to the National Hospital of Burns, Hanoi (day 0). Physical examination showed extensive injuries involving 75% (with a deep injury proportion of 60%) of the total body surface including his face, scalp, chest, abdomen, back, and all extremities. The respiratory system was severely involved. The patient was treated with intravenous fluids, parenteral antibiotics and antifungal drugs, and topical silver sulfadiazine ointment. He underwent surgical measures for debridement and skin transplantation, mechanical ventilation, and dialysis. His hospital course was interspersed with multiple episodes of local and disseminated infections. Wound swab cultures were positive for *Acinetobacter baumannii*, *Pseudomonas aeruginosa*, and *Candida tropicalis*. Blood specimens were taken from the central venous catheter and peripheral veins for culture, and *Stenotrophomonas maltophilia*, *C. tropicalis*, *A. baumannii*, and *P. aeruginosa* were isolated at different time points. *C. tropicalis* fungemia was successfully treated with antifungal drugs as evident by the negative result of blood culture on day 38. However, the patient did not recover from septicemia caused by *P. aeruginosa* and multiple organ failure. The patient’s family insisted on discharge of the patient on day 58 after hospital admittance against urgent medical advice. He died several days after discharge due to multiple organ dysfunction. The anti-infectious management of the patient is depicted in Figure 1.

**Figure 2.**
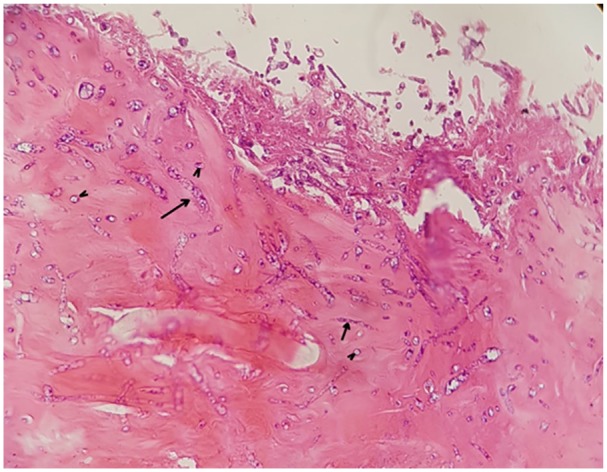
Varicosity hyphae (arrows) and yeast-like structures (arrowheads) of *Fusarium* on slide stained with hematoxylin-eosin (day + 24).

A biopsy taken from the bottom of a burn ulcer on day 24 and stained with hematoxylin-eosin showed proliferating fibrous connective tissue, collagen fibers, fibrinoid necrosis, and newly formed blood vessels. There were abundant varicosities and yeast-like structures indicating fungal infection in the tissue and on the base of the ulcer (Figure 2).

**Figure 3.**
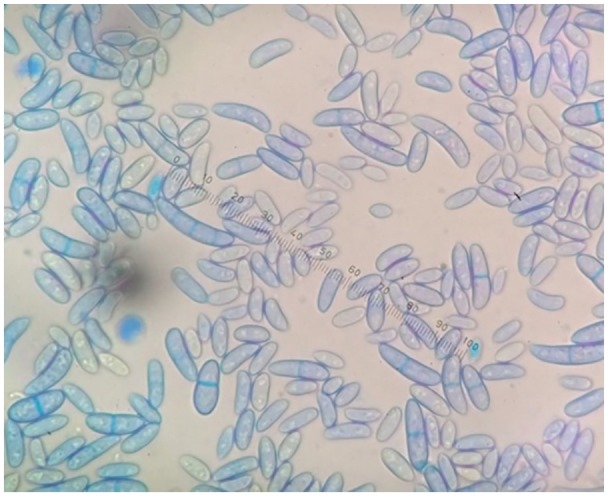
Wide, crescent-shaped macroconidia on microscopic examination of the isolation.

Cultures of the biopsy specimens yielded white colonies and many wide, crescent-shaped macroconidia characteristic of *Fusarium* on microscopic examination (Figure 3).

The isolated strain was subjected to molecular analysis by sequencing the internal transcribed spacer (ITS) region and identified as *F. solani* (ITS sequence submitted to GenBank [MN066126]).

### Susceptibility Testing

Susceptibility testing of the isolation was performed by agar disk diffusion procedure as described previously.^12^ The inhibition zones of amphotericin B and itraconazole were of 8 and 11 mm, respectively, while there was no zone of inhibition to fluconazole, voriconazole, and caspofungin, meaning that the isolation was resistant to all tested drugs.

## Discussion

The patient experienced both local and disseminated infections and the cause of death was multiple organ dysfunction syndrome, which is a direct consequence of sepsis and the leading cause of death in patients with severe burn injuries.^13^

This patient was diagnosed with invasive fusariosis following the detection of *F. solani* in biopsy specimens by culture and histopathological examination.^8^ The diagnosis of fusariosis was suspected when varicose hyphae and yeast-like structures were discovered by histopathological examination^2^ and further confirmed when typical crescent-shaped macroconidia of *Fusarium* spp were detected by microscopy. Molecular species identification was achieved by sequencing of the *ITS1* region; this region is commonly used to identify clinically relevant moulds.^14^ The pathogen was identified as *F. solani*, the most frequent pathogenic agent among *Fusarium* spp. in humans.^2^ Although *Fusarium* is the second most common filamentous fungus associated with burn injury infections, the rate of burn injury infections caused by this fungus is quite rare.^7,15^ *Fusarium* has attained attention due to its angioinvasive property, the virtual absence of viable treatment options,^9^ as well as the high fatality rate among immunocompromised hosts.^16^

The patient was treated with 3 antifungal drugs: caspofungin, fluconazole (for *C. tropicalis*), and voriconazole (for *Fusarium*). Caspofungin and fluconazole were effective against *Candida* but apparently not against *Fusarium.*^17^ Due to the late identification of the causative agent *Fusarium*, specific treatment of fusariosis is usually delayed.^6^ In this case, voriconazole was started 10 days after culturing the biopsy specimen. The high degree of resistance to antifungals of the isolation, in this case, was consistent with other report,^18^ which made managing fusariosis very challenging. Either amphotericin B or voriconazole is commonly applied as the first-line therapy,^9,19^ but voriconazole is not recommended in case of infection with *F. solani.*^2^ At the start of antifungal treatment for fusariosis, the exact species and antifungal susceptibility of the isolation was not yet determined, so voriconazole was a reasonable choice. The outcome of disseminated fusariosis is usually poor, while local infection by *Fusarium* is associated with rather low mortality.^7^ It should be noted that the present patient was infected with many agents including bacteria and fungi, a phenomenon noticed in other reports.^6^ As the patient was infected with several bacteria and fungi, it is not possible to attribute the fatal outcome to one of the organisms.

The case reported here underlines the role of *Fusarium* spp, especially of *F. solani* as an opportunistic pathogen in burn injuries. Improvement in burn injury care and antibiotic treatment regimens have reduced the incidence of bacterial infections; however, the importance of opportunistic fungal infections needs urgently be taken into account. With regard to the risk of fusariosis in patients with burn injuries, this condition should be considered and screened for by histologic and mycologic diagnostics. Molecular analyses for species identification and antifungal susceptibility tests should be applied in order to select antifungal drugs appropriately.
